# Study on Surface Roughness, Morphology, and Wettability of Laser-Modified Powder Metallurgy-Processed Ti-Graphite Composite Intended for Dental Application

**DOI:** 10.3390/bioengineering10121406

**Published:** 2023-12-09

**Authors:** Peter Šugár, Richard Antala, Jana Šugárová, Jaroslav Kováčik, Vladimír Pata

**Affiliations:** 1 Institute of Production Technologies, Faculty of Materials Science and Technology, Slovak University of Technology, J. Bottu 25, 917 24 Trnava, Slovakia; richard.antala@stuba.sk (R.A.); jana.sugarova@stuba.sk (J.Š.); 2 Slovak Academy of Sciences, Institute of Materials and Machine Mechanics, Dúbravská cesta 9, 845 13 Bratislava, Slovakia; jaroslav.kovacik@savba.sk; 3 Department of Production Engineering, Faculty of Technology, Tomas Bata University, Vavrečkova 5669, 960 01 Zlín, Czech Republic; pata@utb.cz

**Keywords:** laser, machining, titanium, composite, powder metallurgy, surface, morphology, roughness, contact angle

## Abstract

In this study, the surface laser treatment of a new type of dental biomaterial, a Ti-graphite composite, prepared by low-temperature powder metallurgy, was investigated. Different levels of output laser power and the scanning speed of the fiber nanosecond laser with a wavelength of 1064 nm and argon as a shielding gas were used in this experiment. The surface integrity of the machined surfaces was evaluated to identify the potential for the dental implant’s early osseointegration process, including surface roughness parameter documentation by contact and non-contact methods, surface morphology assessment by scanning electron microscopy, and surface wettability estimation using the sessile drop technique. The obtained results showed that the surface roughness parameters attributed to high osseointegration relevance (Rsk, Rku, and Rsm) were not significantly influenced by laser power, and on the other hand, the scanning speed seems to have the most prevalent effect on surface roughness when exhibiting statistical differences in all evaluated profile roughness parameters except Rvk. The obtained laser-modified surfaces were hydrophilic, with a contact angle in the range of 62.3° to 83.2°.

## 1. Introduction

In recent years, titanium and its alloys as bone interfacing materials have found relevance primarily in the production of dental implants, fixation screws, and artificial knee or hip joints [1]. Mutual interaction between the human body and a biomedical titanium implant is primarily affected by the implant’s surface ability to osseointegrate, i.e., to create strong anchorage with bone [2,3]. Surface characteristics of the implant, e.g., chemical and phase composition, surface roughness, topography, surface energy, or antibacterial properties, play a crucial role in determining the response of the host biological environment after implantation [4]. In regard to these circumstances, many authors suggest optimization of the morphological and microstructural properties of the implants. The emphasis is laid mostly on micro- and nano-roughness alternation, as well as appropriate space distribution of topographical features in combination with improved thickness of the passivation layer and enhanced surface energy, resulting in strong bioactive and antibacterial properties [5,6]. As a general rule, increased surface roughness is considered to greatly support the initial adhesion, as well as the further proliferation of cells during bone tissue formation, which is explained by the elevated contact area [7,8]. However, while very low or, on the contrary, excessively high roughness can both result in a reduction in cell growth, surfaces with moderate roughness, i.e., Ra parameter in the range of 1 to 2 μm, seem to ensure good osteoblast differentiation [6,8,9,10,11,12]. However, from the point of view of the optimal osseointegration process, the independent effect of the roughness parameter Ra cannot be considered the most influential indicator, and therefore attention should be drawn to surface parameters that offer better insight into the size, shape, and spatial distribution of individual topographical elements, such as the coefficient of kurtosis Rku, the coefficient of skewness Rsk, and the mean width of profile elements Rsm [13,14]. In terms of implant surface topography, cells can exhibit various reactions in contact with surface irregularities, such as depressions, protrusions, pores, or cavities. In regard to a strong osseointegration process, cells need to be equivalent, slightly smaller, or significantly larger than the surrounding topographical features to diminish the local cytoskeletal stress [3,15,16]. However, even topographical features of sizes comparable to the cell dimension that are excessively sharp can hinder cell proliferation and spreading due to the high local stresses on the cell cytoskeleton [17,18]. Thus, the authors believe that in terms of a reliable osseointegration process, the optimal surface profile should be platykurtic (Rku < 3), i.e., consist of a few low and flat protrusions and depressions, with a predominance of depressions (Rsk < 0), divided by relatively wide spacings, crucial for good cell alignment and spreading [19,20]. Furthermore, although the influence of the parameter Rsm has not yet been sufficiently investigated, based on the results of the authors [21], the Rsm should be in the range of 30 to 80 μm to ensure good cellular responses. In addition, the alternation of nanometric roughness provides better biocompatibility, increased surface energy, and very strong anti-adhesion properties toward the numerous bacterial colonies [2,20,22]. Since the rigid membrane of most bacterial cell lines is more susceptible to surface morphology compared to more complex mammalian cells, the nano-roughened surface layer can both support the osteoblastic cell behavior while exhibiting a strong bactericidal effect [23,24].

To ensure better biocompatibility of the titanium implant surface, numerous mechanical (sandblasting, mechanical grinding, polishing), chemical (oxidation, acid etching, anodization), or physical (ion implantation, physical vapor deposition, laser treatment) surface modification methods can be used [25,26]. Among the aforementioned techniques, surface modification by pulsed laser beam, which represents a fast, easily reproducible, and low contamination process, can be utilized for the preparation of surface textures [27,28,29,30,31], isotropic and anisotropic surface structures [32,33], or biocompatible coatings with the appropriate combination of resultant surface integrity and chemical composition [34,35,36,37]. For example, a study [38] revealed that CO_2_ laser treatment helped to significantly enhance cell adhesion and proliferation of MC3T3 and NIH/3T3 cell lines on a TiG5 alloy. On the other hand, Faeda et al. [39] found that titanium implants treated by the ns Nd:YAG laser (Ra = 1.38 ± 0.23 μm) provided a significant increase in the removal torque compared to only mechanically machined specimens. In addition, laser irradiation can help to strengthen the naturally existing passivation layer of Ti, which consists primarily of TiO_2_ particles. Titanium dioxide-based coatings in the form of both anatase and rutile can promote corrosion resistance, antibacterial properties, bone apatite entrapment, and osteoblastic cell responses, as has been demonstrated by several authors [38,40,41,42,43,44,45]. In addition, the enhancement of wettability state and surface energy, which play a major role in interfacial biological responses, helps to rapidly increase protein absorption and improve subsequent cell adhesion, proliferation, and differentiation rates [2,46,47,48]. According to [49,50,51], good osteoblastic cell activity is, alongside the high surface energy and roughness alternation, often correlated with the presence of a high number of hydrophilic hydroxyl and oxidic groups present after the laser treatment.

In summary, stable osseointegration mechanisms of the biomedical implant surface are generally believed to be greatly influenced by surface texture, morphology, roughness, energy, and surface porosity. Nowadays, in terms of the production of reliable implants, more and more authors have begun to utilize porous titanium alloys or Ti-based metal matrix composites (TiMMCs) with a Young’s modulus comparable to that of human bone to prevent the stress-shielding effect. A high Young’s modulus usually causes altered stress distribution on the bone, weakens the bone next to the implant, and causes bone resorption, connection failure, and implant loosening. The porous structure serves as a base for bone tissue interlocking and enhances fluid flow throughout the implant [47,52,53,54,55,56,57,58,59,60,61].

To sum up, research on biocompatible titanium dental implants and processing methods to improve their osseointegration process seems to be essential since many concerns about optimal properties at the interface between the human body and the implant’s surface remain unanswered. This fact, combined with a growing interest in the use of TiMMCs-based complex and cost-effective implant material alternatives with enhanced porosity, prompted the authors to investigate laser micromachining of a Ti-based composite material prepared using a low-temperature powder metallurgy (PM) technique. The newly developed Ti-graphite is a lightweight and highly porous composite material prepared particularly for dental implant application that has a Young’s modulus lower than the widely used Ti Grade 5 alloy. The used technology of compaction represents a low-cost alternative to the wide range of PM techniques, such as the compaction procedure followed by sintering (the press-and-sinter), sintering followed by compacting using hot plastic deformation, direct rolling and extruding of loose powders, the method of hot isostatic pressing (HIP), and MIM (metal injection molding). It gives a material specific microstructure and mechanical properties. To improve the functional characteristics of the Ti-graphite composite surface, the influence of various laser powers and scanning speeds on the resultant surface properties, namely, roughness, morphology, and wettability, were investigated in this study. The modified surface of the porous Ti-graphite composite was examined, assessed, and addressed from the standpoint of use in dental implantology.

## 2. Materials and Methods

### 2.1. Experimental Material

**Figure 1:**
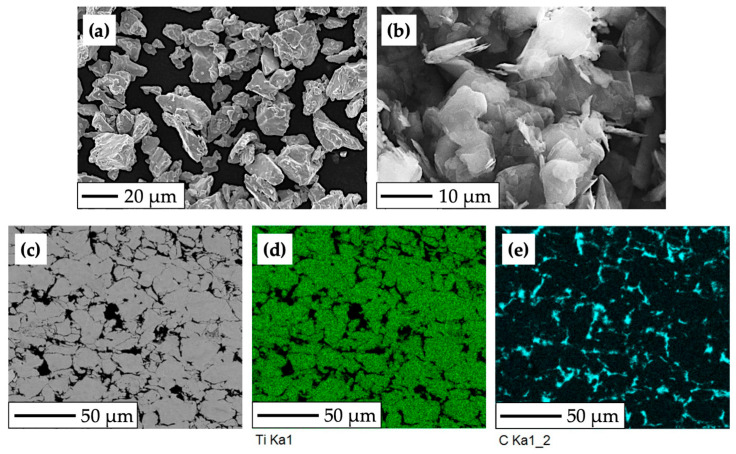
SEM and EBSD analysis of the experimental HDH Ti—graphite composite: (**a**) CP HDH Ti powder at 750× magnification; (**b**) graphite flakes at 2000× magnification; (**c**) structure of the final Ti-graphite composite; (**d**,**e**) phases distribution mapping (Ti—green color, graphite—cyan color).

For the purposes of the experimental procedure, the titanium-based experimental material was composed of CP HDH titanium powder, pictured in Figure 1a (Kimet Special Metal Precision Casting Co., Ltd., Hengshui, China). The particle size of the Ti powder is up to 32 μm, with a sharp, fragment-like shape typical for the hydride–dehydride method. Graphite flakes (Figure 1b), which constitute 15% of the composite’s volume, have an average size of approximately 16 μm, purity of 99.9%, and a flake aspect ratio of 0.1.

Firstly, it was necessary to mechanically mix the two components using turbulent dry mixing for 30 min to uniformly distribute particles before the compaction process. Subsequently, cold isostatic pressing (CIP) at a pressure of 200 MPa was used to prepare green compacts (molding porosity was in the range of 32 to 40%), followed by hot vacuum pressing (HVP) at a temperature of 450 to 470 °C and pressure of 500 MPa. The resulting porosity and density, determined from weighing and volume measurement, were approximately 2.44 ± 0.15% and 4.1 to 4.15 g·cm^−3^.

The use of a low-temperature powder metallurgy method led to the formation of a titanium composite structure with uniformly distributed graphite particles (Figure 1c) bonded by mechanical action, which resulted in increased porosity and relatively low density, weight, and Young’s modulus (E = 96.83 GPa, determined by nanoindentation measurement and the Oliver–Pharr method). Figure 1c depicts the microstructure of the experimental material, with brighter areas indicating compacted grains of HDH Ti powder and the darker areas indicating the presence of graphite flakes with high carbon content.

### 2.2. Surface Modification Process

Laser surface modification is based on the material’s surface irradiation when the energy is concentrated over a minimal range. The temperature of the processed material substantially increases, followed by material melting and evaporation. With an increase in energy, the recoil force in the molten material increases and ejects it to the edge of the created liquid reservoir. Solidified material gets rougher as a result of the liquid phase solidification.

In this study, 10 different square-shaped surfaces labeled P1–P5 (Group P) and V1–V5 (Group V) were laser machined (Table 1). Prior to the machining process, the samples were firstly cut by electric discharge machining, then grounded with P1200 (15.3 µm) Buehler CarbiMet emery paper, and cleaned in an ultrasonic cleaner with distilled water and bioethanol for 15 min (30 °C). Samples were finally dried with a stream of hot air.

**Figure 2:**
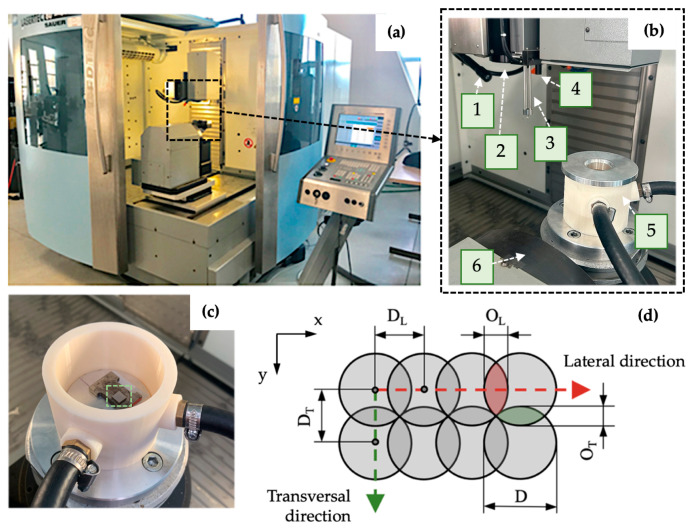
Experimental setup: (**a**) laser machining center Lasertec 80 Shape; (**b**) machine workspace; (**c**) shielding system with non-irradiated specimen marked by a green-dashed square; (**d**) scheme of pulse mode. 1—lighting, 2—laser beam output, 3—measuring probe, 4—CCD camera, 5—Ar shielding system, 6—worktable kinematics, D—spot diameter, D_L_—pulse-to-pulse distance, O_L_—lateral overlapping, D_T_—transversal spacing, O_T_—transversal overlapping.

The final dimensions of the prepared specimen were 7 mm × 7 mm × 4 mm. The surface modification of the samples was carried out on a 5-axis Lasertec 80 Shape machining center (DMG Mori GmbH., München, Germany) (Figure 2a,b), utilizing the pulsed Yb-doped fiber laser working at 1064 nm wavelength, a constant pulse duration of 120 ns, and a laser spot diameter of 50 μm. To limit undesirable chemical products during machining, samples were placed in a shielding chamber with a constant flow of Ar gas through two inlet channels (Figure 2c).

Laser micromachining was performed using the crosshatching strategy when a laser beam traverses an irradiated surface in two perpendicular directions. The amount of heat delivered to the material can be regulated by average output power P, laser spot diameter D, laser pulse frequency f, and scanning speed vs. (these two parameters determine pulse-to-pulse distance in lateral direction D_L_ and transversal spacing D_T_) (Figure 2d).

Formula (1) can be used to determine the total amount of transferred energy E_T_ delivered to the irradiated area, which is given by the pulse energy of the laser beam E_P_ and the summary of incident pulses of the laser beam in one place N expressed by Equation (2), where the parameter D_L_ can be calculated according to Equation (3) [20].

(1) $$E_{T}=E_{P}\;\times \;N\;$$

(2) $$N=\frac{D^{2}}{D_{L}\;\times \;D_{T}}$$

(3) $$D_{L}=\frac{v_{s}}{f}$$

### 2.3. Surface Characterization

In terms of surface integrity, primarily indicators of field surface roughness, namely Ra, Rp, Rv, Rz, Rsk, and Rku, as well as the spacing parameter Rsm and parameters related to Abbot–Firestone curves, were tested by the contact gauge profilometer Mitutoyo SJ 210 (Mitutoyo Europe GmbH, Neuss, Germany) according to [62]. The 3D optical surface profiler Zygo New View 8000 (Zygo Corporation, Middlefield, OH, USA) was used for the identification of the surface texture by the areal method according to [63].

The resulting surface topography and morphology, as well as the presence of surface defects (pores, voids, cracks, and spatter), were assessed by SEM analysis using the high-resolution scanning electron microscope JEOL JSM 7600F (JEOL Ltd., Tokyo, Japan). All modified surfaces were observed at magnifications from 25 to 5000× in the secondary electron imagining regime by U = 15 keV, I = 1.0 nA, and WD = 15 mm.

The static contact angle (CA) measurements were applied to analyze the laser-modified surface wettability using See System E (Advex Instruments, s. r. o., Brno, Czech Republic).

The measurements were conducted more than 30 days after laser treatment, applying deionized water droplets with a volume of 10 μL with 3 repetitions of the tests on each surface. The samples were cleaned with deionized water and dried before each measurement. The CA reading was started 5 s after the droplet was placed on the surface. The droplet height h and width d were documented, and the CA was calculated according to Equation (4).

(4) $$\theta =2\;\tan^{-1}\frac{2h}{d}$$

### 2.4. Statistical Analysis

To assess differences in roughness and wettability of experimental surfaces, a one-way analysis of variance (ANOVA) was performed using Minitab v.17 software (Minitab, LLC, State College, PA, USA). In the case of significant differences, the Tukey HSD post hoc test was applied. The levels of significance at 95% (α = 0.05) and 99% (α = 0.01) were chosen.

## 3. Results and Discussion

### 3.1. Surface Roughness Measurement Results

**Figure 3:**
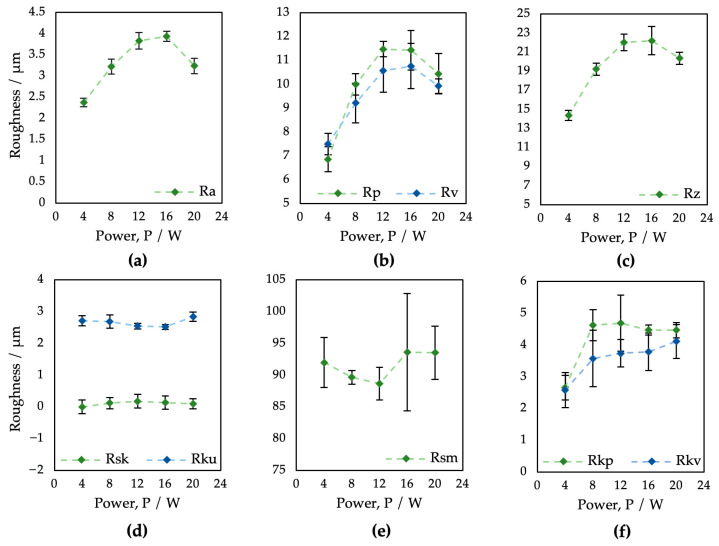
Evaluation of profile roughness parameters of surfaces P1–P5 after laser treatment applying different values of laser power: (**a**) Ra; (**b**) Rp and Rv; (**c**) Rz; (**d**) Rsk and Rku; (**e**) Rsm; (**f**) Rpk and Rvk.

The results of the surface roughness evaluation depending on the laser output power variation are summarized in Table 2 and Figure 3. Studied were field parameters of the surface roughness as follows: arithmetic mean high Ra, mean peak height Rp and mean pit depth Rv, total height Rz, skewness Rsk, kurtosis Rku, mean profile element spacing Rsm, as well as reduced peak height Rpk and reduced pit depth Rvk.

In the case of the arithmetic mean height Ra (Figure 3a), on all samples, the surface roughness ranged from 2.37 to 3.94 μm. Regarding the output power, the Ra parameter values tended to increase with increasing laser power; however, the higher power of 16 W (E_T_ = 2 mJ) caused a roughness reduction. As can be seen, the Rp, Rv, and Rz parameters also decreased via laser power exceeding 16 W (E_T_ = 2 mJ). In the case of parameters Rp and Rv (Figure 3b), only surface P1 showed the presence of deeper pits over peaks, probably due to a combination of low material scattering and initial surface porosity caused by the PM process. In terms of laser-induced skewness (Figure 3d), all resultant surfaces, except P1, exhibited a predominance of larger peaks (Rsk > 0). According to the kurtosis results (Figure 3d), all surfaces are platykurtic, i.e., less rugged with a relatively low number of hills and dales (Rku < 3). Interestingly, there was almost no impact of the laser power on the skewness Rsk, kurtosis Rku, or mean element spacing Rsm.

**Figure 4:**
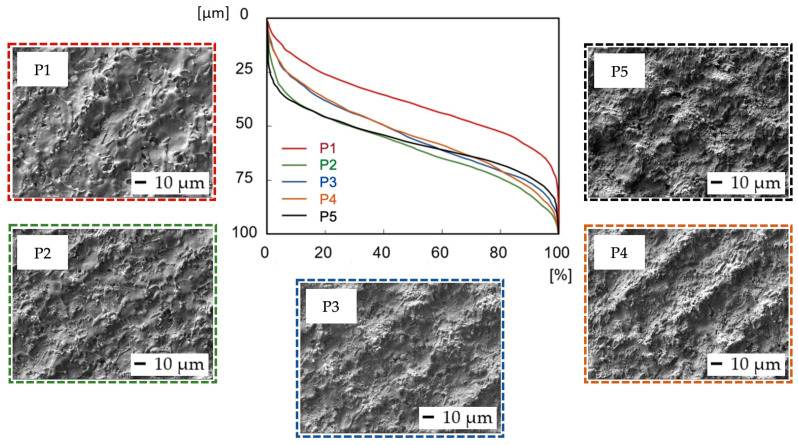
Abbott–Firestone curves after laser irradiation of samples P1–P5.

Abbott–Firestone curves (Figure 4) show the reduced peak height Rpk and reduced pit depth Rvk that are crucial from the point of view of predicting the initial properties after implantation. To diminish initial wear and alter the surface’s ability to absorb fluids, fuller profile curves with small slopes, low Rpk, and high Rvk are generally preferred. On all the other surfaces, whose shapes are very similar, the resultant Rpk exceeded the Rvk values.

The laser treatment with a variation in scanning speed was performed by using the same level of pulse energy (0.2 mJ). Despite the constant level of output power, the amount of transferred energy remained in a relatively similar range from 0.4 to 2 mJ because the variation in pulse-to-pulse distances from 25 to 125 μm strongly affects the summary of incident pulses of the laser beam in one place. An increase in scanning speed should, in theory, reduce the level of transferred energy and thus diminish the impact of the laser beam on surface roughness.

**Figure 5:**
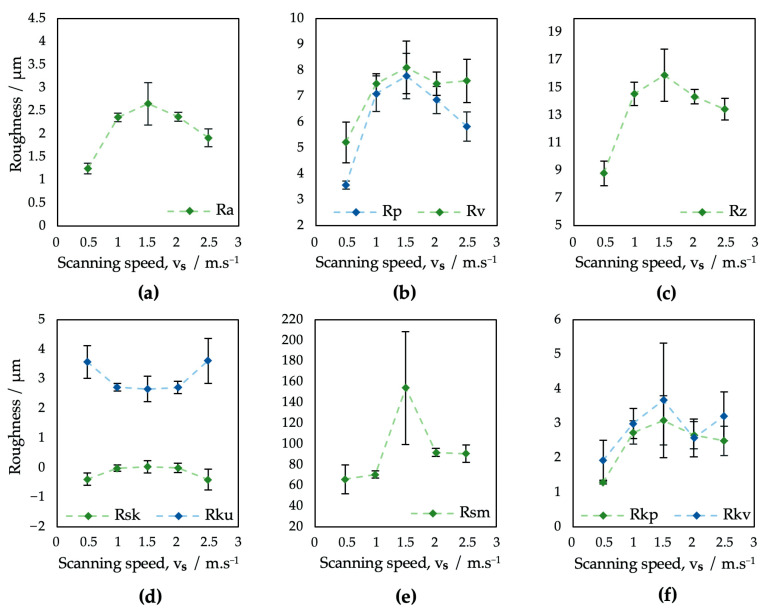
Evaluation of surface roughness parameters of samples V1–V5: (**a**) Ra; (**b**) Rp and Rv; (**c**) Rz; (**d**) Rsk and Rku; (**e**) Rsm; (**f**) Rpk and Rvk.

The field parameters of the surface roughness of samples treated using different laser beam scanning speeds are shown in Table 3 and Figure 5.

In terms of the roughness parameter Ra (Figure 5a), surfaces V1–V5 exhibited results in the range from 1.25 to 2.66 μm. The increase in the scanning speed of the laser beam did not result in an unambiguous trend of monotony.

While on surface V1 the overlap of laser pulses of 50% resulted in the overall lowest level of Ra, for the other four analyzed surfaces, where no overlapping was applied, the Ra reached higher values. According to study [64], the surface roughness greatly varies with pulse-to-pulse overlap because of its effect on crater and rim geometry.

The monotony of all the tested roughness parameters on surfaces V1–V5 tends to greatly change at a scanning speed of 1500 mm·s^−1^, which might be caused by a combination of a higher pulse-to-pulse distance and relatively low laser power.

In addition, the kurtosis of the obtained surfaces seems to vary between 2.67, corresponding to the platykurtic profile, and 3.62, corresponding to the leptokurtic, i.e., a more rugged surface profile (Figure 5d). Furthermore, the results of surface skewness (Figure 5d) tend to vary between 0.40 and 0.11. The scanning speed differences also resulted in a bigger fluctuation of the mean profile element spacing Rsm, which varied in the range from 66.08 to 92 μm (Figure 5e), with an extreme value in the case of surface V3, where an Rsm = 154.14 μm was documented.

**Figure 6:**
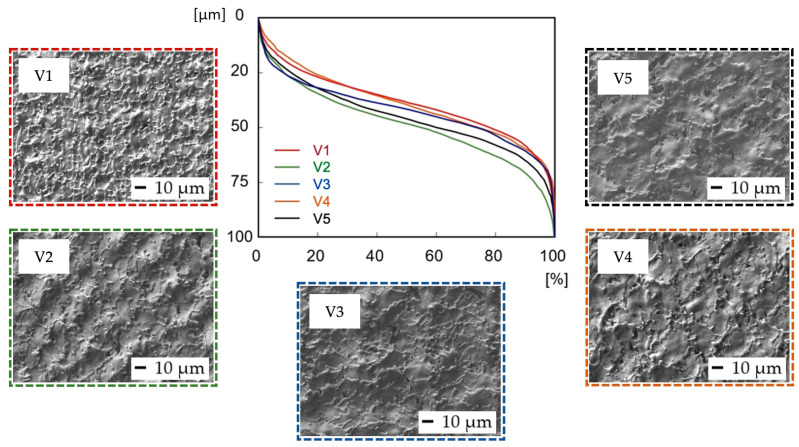
Evaluation of Abbott–Firestone curves after laser irradiation of samples V1–V5.

In terms of initial wear and fluid absorbance (Figure 6), surfaces V1–V5 exhibited a very similar, full surface profile, comparable to the profile of sample P1, prepared with the same level of output power (4 W) (Figure 4). The Rvk parameter on surfaces V1–V5 exceeded the Rpk in all cases, which might positively influence implant surface wettability towards body fluids. The dimensions of the peaks and pits are smaller compared to the P1–P5 samples.

While the lower output power of the laser beam has limited the occurrence of visible surface textures, higher output power levels result in the formation of relatively regular square-shaped surface textures with the presence of various nanosized hydrodynamic features, but with only a shallow Rsm parameter range of 88.68 to 93.62 μm. A variation in scanning speed did not induce nano-geometrical feature formation and helped to induce a wide interval of Rsm values starting at 66.08 μm that covered the recommended interval by [21]. For these circumstances, the obtained spacing parameter Rsm range from 66 to 94 µm seems to be sufficient, because according to studies [13,51,65] a presence of grooves created by topographical features smaller than 100 µm has a strong effect on a single cell’s behavior and can help to improve cell adhesion and its homogenous orientation.

**Figure 7:**
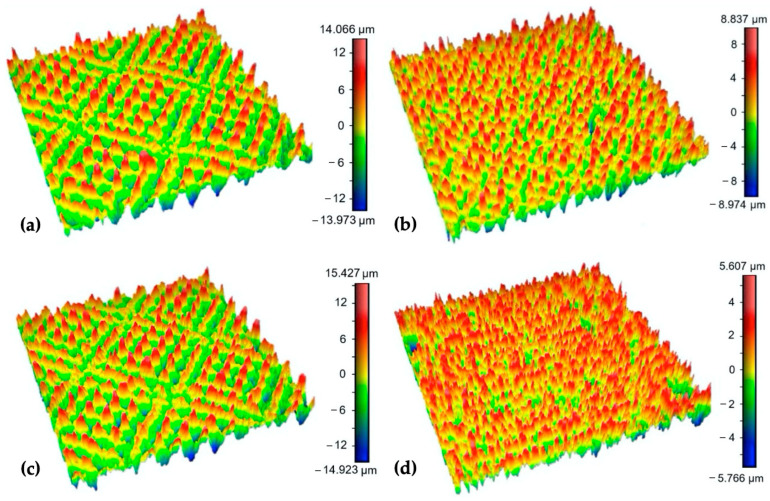
3D maps of the surfaces: (**a**) P2; (**b**) V2; (**c**) P4; (**d**) V1 (surface area 0.8 × 0.8 mm).

The experimental results showed that surfaces processed using the same values of the transferred energy E_T_ while different combinations of laser power and scanning speed were used exhibited different surface morphologies and surface roughness parameters. It is documented in Figure 7, which shows the 3D maps of the surfaces P2 and V2 processed using E_T_ = 1 mJ and the surfaces P4 and V1, where E_T_ = 2 mJ was employed. While E_T_ = 2 mJ on surface V1 resulted in a low Rsm parameter (66.08 μm), presumably because of the low scanning speed and pulse-to-pulse overlap of 50%, E_T_ = 2 mJ in the case of surface P4, prepared via higher output power and scanning speed, exhibited an Rsm parameter equal to 93.62 μm. Also, a very high difference can be seen between the ratio of the dale void volume (Vvv) to peak material volume (Vmp), where it reached a value of 2.1 for surface P4 and 4.6 for surface V1.

Based on the one-way ANOVA results, it can be stated that for the variation in the output power values, the parameters Ra, Rp, Rv, Rz, Rpk, and Rvk are all statistically different; however, the parameters Rsk, Rku, and Rsm, which are attributed to high osseointegration relevance, are not statistically different at the *p*-value level of neither 0.01 nor 0.05 (Table 4).

The variation in scanning speed exhibited statistically significant differences in all evaluated roughness parameters except the Rvk, which just slightly exceeded the *p*-value of 0.05 (Table 5).

The Tukey HSD post hoc test has not confirmed statistically significant differences for every combination of analyzed surfaces, e.g., almost identical Ra roughness parameter findings were found for the pairings of P2–P5 and P4–P3. On the other hand, in terms of the spacing parameter Rsm, the samples V1–V5 may have statistically significant differences only conditionally due to the presence of sample V3 (E_T_ = 0.67 mJ) with very specific surface properties.

### 3.2. Scanning Electron Microscopy (SEM) Observation Results (Variation of Output Laser Power)

**Figure 8:**
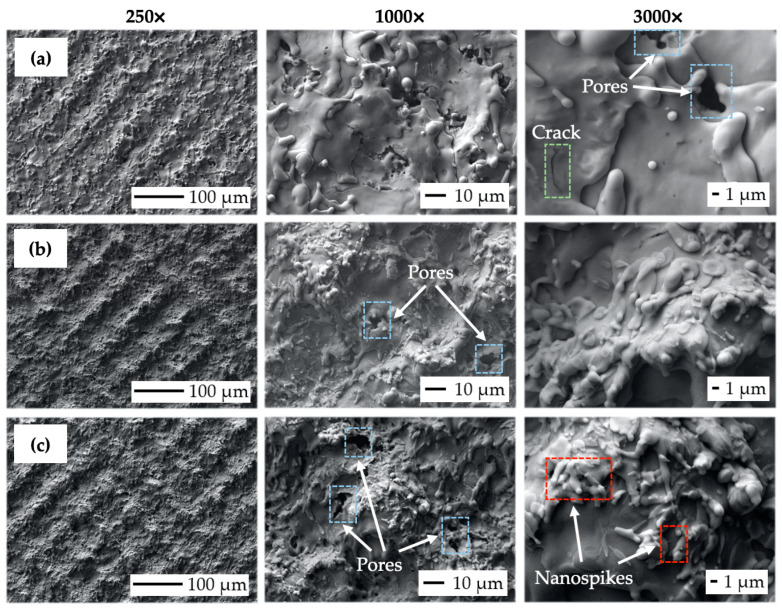
Scanning electron microscopy (SEM) micrographs of the machined surfaces: (**a**) surface P1 (P = 4 W, E_T_ = 0.5 mJ); (**b**) surface P3 (P = 12 W, E_T_ = 1.5 mJ); (**c**) surface P5 (P = 20 W, E_T_ = 2.5 mJ).

Figure 8 depicts SEM micrographs of the laser-treated samples at different levels of output laser power at 250×, 1000×, and 3000× magnification. It can be observed that the crosshatching strategy of laser beam movement resulted in the formation of a wavy and fragmented layer of remelted and subsequently solidified material on all the machined surfaces. In terms of final surface morphology, while a lower amount of transferred energy, corresponding to the lower level of laser beam power, has limited the occurrence of visible surface texture (Figure 8a), a higher amount of transferred energy, corresponding to the higher output power levels, results in the formation of relatively regular square-shaped surface textures with the presence of various nanosized hydrodynamic features (Figure 8b,c).

While nanosized spikes, present on surface P5 (Figure 8c), could potentially enhance antibacterial properties, the presence of microsized slot-like surface morphology could, on the other hand, utilize the contact guidance phenomena studied by [30,66,67] to improve cell adhesion, proliferation, and subsequent differentiation. It can be seen that the increase in the transferred energy level given by the rise in output power caused the alternation of surface roughness, material spattering, and pore initiation (blue color in Figure 8). The formation of a nano-geometric particle of melted, scattered, and resolidified material was found to be highly correlated with the laser energies used. This relation was also observed by [68,69], who also stated that droplet formation in the size range of 1 to 2 μm is suitable for better migration of donut-shaped red blood cells.

The crack initiation seems to not increase with variations in laser power levels (green color in Figure 8). According to study [69], the rise in the output power of a pulsed laser beam tends to greatly enhance droplet formation, which is in the size range of 1 to 2 μm and is suitable for better migration of donut-shaped red blood cells.

#### Scanning Electron Microscopy (SEM) Observation Results (Variation of Scanning Speed)

**Figure 9:**
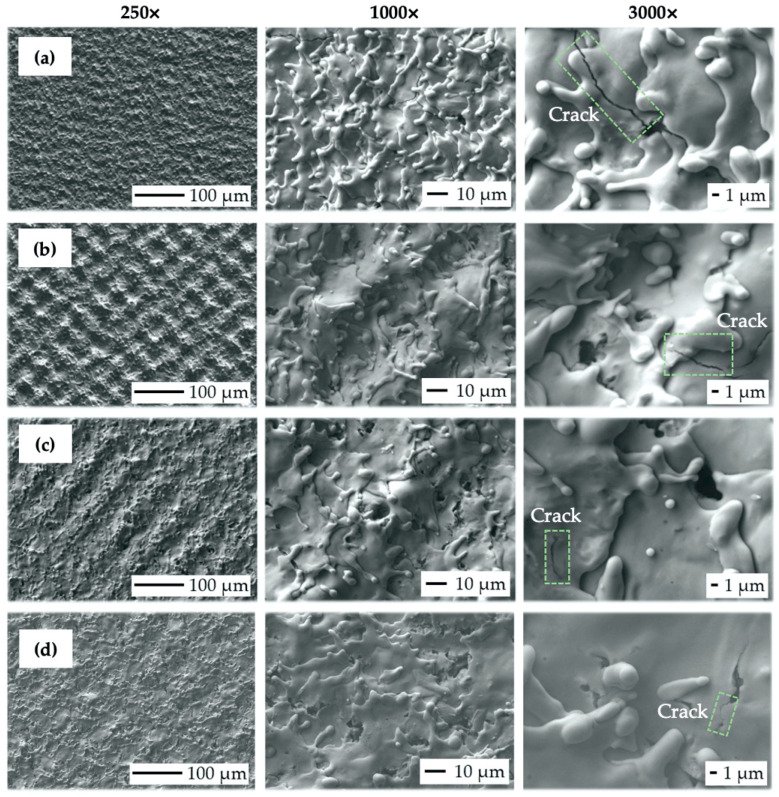
Scanning electron microscopy (SEM) micrographs of the machined surfaces: (**a**) V1 (vs. = 500 mm·s^−1^, E_T_ = 2 mJ); (**b**) V2 (vs. = 1000 mm·s^−1^, E_T_ = 1 mJ); (**c**) V4 (vs. = 2000 mm·s^−1^, E_T_ = 0.5 mJ); (**d**) V5 (vs. = 2500 mm·s^−1^, E_T_ = 0.4 mJ).

It can be observed that the crosshatching strategy in the case of scanning speed variation resulted in a variety of wavy topographical structures, probably due to the more diverse movement of the laser beam over the modified surfaces (Figure 9).

In terms of final surface morphology, while moderate levels of transferred energy (E_T_ = 0.5 to 1 mJ), corresponding to the scanning speed level from 1000 to 2000 mm·s^−1^ (Figure 9b,c), were resulting in the formation of relatively regular square-shaped surface textures, a higher transferred energy level (E_T_ = 2 mJ), corresponding to the lowest scanning speed of 500 mm·s^−1^ and 50% laser spot overlap (Figure 9a), was inducing irregular surface textures.

On the contrary, sample V5 (Figure 9d), prepared with the highest scanning speed of 2500 mm·s^−1^ (E_T_ = 0.4 mJ), also exhibited irregular surface morphology with almost invisible laser beam traces, which can be considered insufficient in terms of cell contact guidance phenomena.

In addition, an increase in scanning speed resulted in less vivid material spattering and diminished crack initiation (green color in Figure 9). Interestingly, the presence of nanosized hydrodynamic effects was eliminated even though the transferred energy range remained very similar to the previous test.

### 3.3. Wettability Test

**Figure 10:**
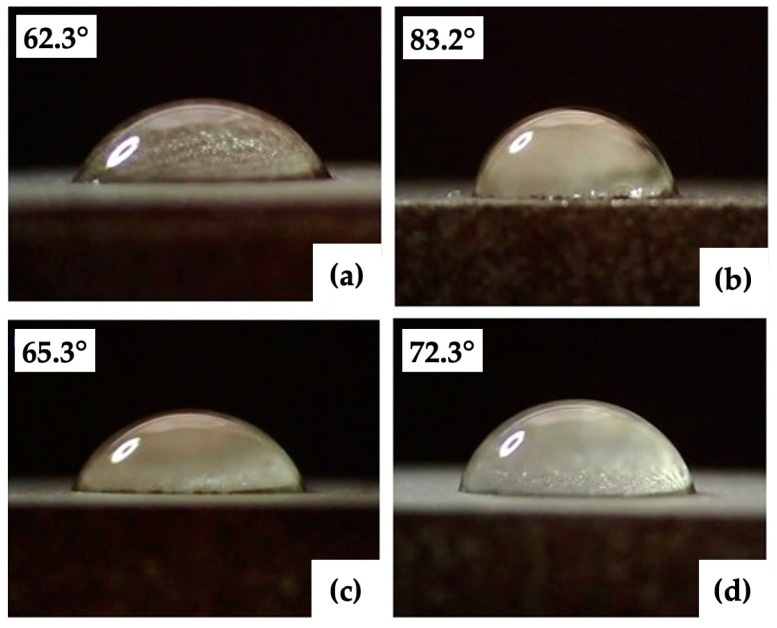
Minimal and maximal contact angles: (**a**,**b**) group of the surfaces P (P3, P5); (**c**,**d**) group of the surfaces V (V1, V5).

**Figure 11:**
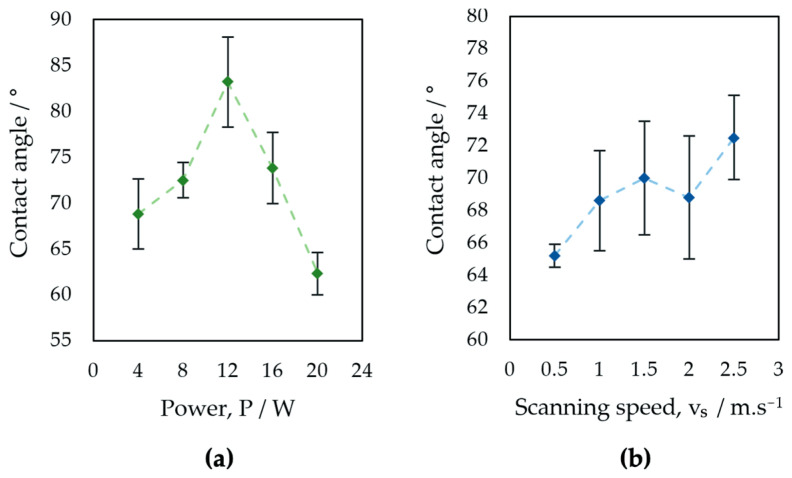
Evaluation of contact angle: (**a**) surfaces P1–P5, (**b**) surfaces V1–V5.

The results of the surface wettability assessment are summarized in Table 6 and Table 7 and Figure 10 and Figure 11. All evaluated surfaces were hydrophilic, with a contact angle in the range of 62.3° to 83.2°, which indicates hydrophilic surfaces have the potential to promote protein adhesion and osteoblastic cell attachment [13,70,71]. An increase in contact angle was documented with increasing laser power up to 12 W, followed by decreasing the contact angle value for surfaces P3 and P4 machined with 16 and 20 W. Statistical analysis of the surfaces of group P showed statistically significant differences in the contact angles of surfaces P2, P3, and P5 for α = 0.05 as well as α = 0.01.

For samples of group V, an increase in contact angle is associated with increasing laser beam scanning speed. A small decrease in the contact angle was observed in the case of surface V4, machined with a laser beam scanning speed of 2 m·s^−1^. The contact angles documented on the surfaces of Group V did not show statistically significant differences for α = 0.05 (Table 8).

The obtained results agree with the theory that the contact angle of the 30-day-old titanium surface ranges from 60° to 120° [72,73,74]. The relationship between laser power and wettability is similar to the relationship between laser power and all surface roughness parameters evaluated in the experiment. The similarity of the relationships between the scanning speed and the contact angle and between the scanning speed and surface roughness was not documented. The measured contact angles are partially influenced by the dual-scale micro/nano surface structures of the evaluated surfaces, which can initiate the transition toward hydrophobicity, as has been confirmed by previous studies [75,76].

In addition to the surface morphology and roughness, the contact angle of the laser-machined surfaces is also influenced by the surface chemistry determined by the used laser processing parameters. According to [22,77,78,79], the higher surface roughness resulted in a lower value of the contact angle, but with surface oxidation and organic contamination over time, the contact angle increased. The phase composition of the Ti-graphite composite after laser treatment was studied by the authors in [35]. Two types of oxides were detected on the surface of samples after laser ablation in the Ar shielding gas, namely, cubic TiO and rhombohedral Ti_2_O_3_, with a content of 50% and 60%, respectively. Additionally, it can be supposed that due to the microstructural changes and oxidation processes in the remelted layer, the titanium surface after laser modification exhibits an increase in hardness. The laser surface fusion may also result in the formation of a thermally affected area with residual tensile stress. Lowering the tensile residual stress is essential for an increase in surface energy and, thus, a positive impact on cell activity because cells tend to adhere preferentially to stress-free areas [80].

To summarize, the surface hydrophilicity acquired in this study may be expected to be promoting cells’ growth and osteodifferentiation [45,69]. Furthermore, as has been confirmed by previous studies [81,82], the effect of surface aging can potentially lead to either a hydrophobic state, which is generally useful against hydrophilic bacterial strains, or a superhydrophobic state, where bacterial adhesion is limited due to air bubble entrapment typical for a Cassie–Baxter state.

## 4. Conclusions

In this study, the Ti-graphite composite that had undergone low-temperature powder metallurgical processing was treated using various levels of output power and the scanning speed of the nanosecond laser, followed by surface morphology, roughness, and wettability studies. The following conclusions might be drawn from the experimental and statistical analyses:

(1) The moderate level of laser beam energies (0.5 to 1 mJ) helps to induce the presence of regular surface morphology with sufficient spatial distribution, while the higher level of transferred energy (2 to 2.5 mJ) seems to induce irregular surface texture, as well as the formation of nano-geometric features with potential antibacterial effects.
(2) The slot-like morphology observed on the evaluated surfaces might potentially exhibit contact guidance for cultivated cells.
(3) The field surface characteristics that are attributed to high osseointegration relevance (Rsk, Rku, and Rsm) are not statistically different with variation in output power. Interestingly, variation in scanning speed seems to have the most prevalent effect on surface topography when exhibiting statistical differences in all profile roughness parameters except for the Rvk at *p*-value levels of 0.05 and 0.01.
(4) Applying the same values of the transferred energy while different combinations of laser power and scanning speed were used exhibited different surface morphologies and surface roughness parameters.
(5) An increase in contact angle was documented with increasing laser power up to 12 W, followed by decreasing the contact angle for higher laser power values. An increase in contact angle with increasing laser beam scanning speed was observed, but the changes in contact angle were not statistically significant.

## Figures and Tables

**Table 1:** Parameters of laser treatment.

Surface	Output Power (W)	v_s_ (mm·s^−1^)	D_L_ (μm)	E_P_ (mJ)	E_T_ (mJ)
P1	4	2000	100	0.2	0.5
P2	8			0.4	1
P3	12			0.6	1.5
P4	16			0.8	2
P5	20			1	2.5
V1	4	500	25	0.2	2
V2		1000	50		1
V3		1500	75		0.67
V4		2000	100		0.5
V5		2500	125		0.4
Constant parameters: Pulse frequency: f = 20 kHz; Transversal spacing: D_T_ = 10 μm Laser beam movement strategy: cross-hatching; Number of ablated layers: 2; Argon flow rate: 20 L.min^−1^					

**Table 2:** Field parameters of the surface roughness of samples P1–P5.

Surface	P1		P2		P3		P4		P5	
Parameter	Mean	SD	Mean	SD	Mean	SD	Mean	SD	Mean	SD
Ra (μm)	2.37	0.10	3.22	0.18	3.83	0.20	3.94	0.12	3.24	0.18
Rp (μm)	6.86	0.52	10.00	0.45	11.48	0.32	11.44	0.83	10.45	0.85
Rv (μm)	7.49	0.45	9.21	0.83	10.57	0.90	10.77	0.94	9.93	0.31
Rz (μm)	14.35	0.53	19.22	0.64	22.05	0.87	22.20	1.48	20.38	0.63
Rsk (-)	0.00	0.16	0.12	0.20	0.18	0.09	0.14	0.07	0.10	0.15
Rku (-)	2.72	0.21	2.69	0.18	2.55	0.21	2.52	0.21	2.84	0.16
Rsm (μm)	92.00	3.94	89.68	1.09	88.68	2.57	93.62	9.22	93.54	4.19
Rkp (μm)	2.66	0.39	4.62	0.49	4.69	0.88	4.47	0.16	4.47	0.24
Rkv (μm)	2.58	0.55	3.58	0.89	3.74	0.44	3.79	0.59	4.11	0.54

**Table 3:** Field parameters of the surface roughness of samples V1–V5.

Surface	V1		V2		V3		V4		V5	
Parameter	Mean	SD	Mean	SD	Mean	SD	Mean	SD	Mean	SD
Ra (μm)	1.25	0.12	2.36	0.09	2.66	0.46	2.37	0.10	1.92	0.19
Rp (μm)	3.57	0.16	7.10	0.68	7.79	0.87	6.86	0.52	5.84	0.57
Rv (μm)	5.22	0.79	7.49	0.39	8.12	1.02	7.49	0.45	7.60	0.83
Rz (μm)	8.79	0.88	14.55	0.85	15.90	0.89	14.35	0.53	13.44	0.78
Rsk (-)	−0.38	0.21	−0.01	0.11	0.04	0.21	0.00	0.16	−0.40	0.35
Rku (-)	3.58	0.55	2.72	0.12	2.67	0.43	2.72	0.21	3.62	0.76
Rsm (μm)	66.08	13.91	70.78	3.29	154.14	54.34	92.00	3.94	90.82	8.31
Rkp (μm)	1.29	0.04	2.74	0.34	3.09	0.71	2.66	0.39	2.50	0.42
Rkv (μm)	1.93	0.58	3.00	0.44	3.67	1.66	2.58	0.55	3.21	0.71

**Table 4:** One-way ANOVA results—roughness of surfaces P1–P5.

Roughness Parameter	F-Value	*p*-Value	R^2^
Ra	76.62	0.000 *	93.87
Rp	44.77	0.000 *	89.95
Rv	16.35	0.000 *	76.58
Rz	63.50	0.000 *	92.70
Rsk	1.07	0.398	17.61
Rku	2.32	0.093	31.65
Rsm	1.00	0.430	16.69
Rpk	14.75	0.000 *	74.68
Rvk	4.40	0.010 *	46.83

* At least one mean is different for α = 0.05 and α = 0.01.

**Table 5:** One-way ANOVA results—roughness of surfaces V1–V5.

Roughness Parameter	F-Value	*p*-Value	R^2^
Ra	27.22	0.000 *	84.48
Rp	36.66	0.000 *	88.00
Rv	11.68	0.000 *	70.02
Rz	31.25	0.000 *	86.21
Rsk	5.00	0.006 *	50.01
Rku	5.40	0.004 *	51.91
Rsm	9.54	0.000 *	65.61
Rpk	12.36	0.000 *	71.20
Rvk	2.65	0.063	34.65

* At least one mean is different for α = 0.05 and α = 0.01.

**Table 6:** Wettability test results—surfaces P1–P5.

Surface	P1		P2		P3		P4		P5	
Parameter	Mean	SD	Mean	SD	Mean	SD	Mean	SD	Mean	SD
CA (^o^)	68.9	3.8	72.5	2.0	83.2	4.9	73.8	3.9	62.3	2.3

**Table 7:** Wettability test results—surfaces V1–V5.

Surface	V1		V2		V3		V4		V5	
Parameter	Mean	SD	Mean	SD	Mean	SD	Mean	SD	Mean	SD
CA (^o^)	65.3	0.7	68.6	3.1	70.0	3.5	68.8	3.8	72.5	2.6

**Table 8:** One-way ANOVA results—contact angle of surfaces P1–P5 and V1–V5.

CA (^o^)	F-Value	*p*-Value	R^2^
P1–P5	13.79	0.000 *	84.66
V1–V5	2.38	0.121	48.76

* At least one mean is different for α = 0.05 and α = 0.01.

## Data Availability

The data presented in this study are available on request from the corresponding author.
